# Mining Autoimmune-Disorder-Linked Molecular-Mimicry Candidates in *Clostridioides difficile* and Prospects of Mimic-Based Vaccine Design: An In Silico Approach

**DOI:** 10.3390/microorganisms11092300

**Published:** 2023-09-12

**Authors:** Saleh Alshamrani, Mutaib M. Mashraqi, Ahmad Alzamami, Norah A. Alturki, Hassan H. Almasoudi, Mohammed Abdulrahman Alshahrani, Zarrin Basharat

**Affiliations:** 1 Department of Clinical Laboratory Sciences, College of Applied Medical Sciences, Najran University, Najran 61441, Saudi Arabia; saalshamrani@nu.edu.sa (S.A.); hhalmasoudi@nu.edu.sa (H.H.A.); maalshahrani@nu.edu.sa (M.A.A.); 2 Clinical Laboratory Science Department, College of Applied Medical Science, Shaqra University, AlQuwayiyah 11961, Saudi Arabia; aalzamami@su.edu.sa; 3 Clinical Laboratory Science Department, College of Applied Medical Science, King Saud University, Riyadh 11433, Saudi Arabia; noalturki@ksu.edu.sa; 4 Alpha Genomics (Private) Limited, Islamabad 45710, Pakistan

**Keywords:** *Clostridioides difficile*, molecular mimicry, autoimmunity, vaccine design, bioinformatics, immune response, docking, pathogenesis

## Abstract

Molecular mimicry, a phenomenon in which microbial or environmental antigens resemble host antigens, has been proposed as a potential trigger for autoimmune responses. In this study, we employed a bioinformatics approach to investigate the role of molecular mimicry in *Clostridioides difficile*-caused infections and the induction of autoimmune disorders due to this phenomenon. Comparing proteomes of host and pathogen, we identified 23 proteins that exhibited significant sequence homology and were linked to autoimmune disorders. The disorders included rheumatoid arthritis, psoriasis, Alzheimer’s disease, etc., while infections included viral and bacterial infections like HIV, HCV, and tuberculosis. The structure of the homologous proteins was superposed, and RMSD was calculated to find the maximum deviation, while accounting for rigid and flexible regions. Two sequence mimics (antigenic, non-allergenic, and immunogenic) of ≥10 amino acids from these proteins were used to design a vaccine construct to explore the possibility of eliciting an immune response. Docking analysis of the top vaccine construct C2 showed favorable interactions with HLA and TLR-4 receptor, indicating potential efficacy. The B-cell and T-helper cell activity was also simulated, showing promising results for effective immunization against *C. difficile* infections. This study highlights the potential of *C. difficile* to trigger autoimmunity through molecular mimicry and vaccine design based on sequence mimics that trigger a defensive response.

## 1. Introduction

Molecular mimicry refers to the phenomenon in which microbial or environmental antigens share structural or sequence similarities with host antigens [1]. This similarity can lead to a cross-reactive immune response, in which the immune system mistakenly targets self-tissues, resulting in autoimmune disorders [2]. The onset of autoimmune disorders due to molecular mimicry by pathogenic proteins or antigens presents an intriguing research area that investigates the possible connection between microbial infections and the onset of autoimmune responses [3,4]. Understanding the mechanisms by which molecular mimicry contributes to autoimmune disorders is crucial for developing targeted therapies and preventive strategies [5,6,7]. Numerous studies have investigated the molecular mimicry between human proteins associated with autoimmune disorders and pathogen-derived proteins [8,9,10,11,12,13]. These investigations have revealed several mechanisms that contribute to the initiation and perpetuation of autoimmune responses [14,15,16]. Specific pathogen proteins have been found to share sequence motifs with host proteins involved in autoimmune disorders, enabling the activation of autoreactive T cells [14,17]. *Streptococcus pyogenes* has been implicated in autoimmune diseases, such as rheumatic fever [18], glomerulonephritis [19], and multiple sclerosis [20]. Epstein–Barr virus has been connected with molecular-mimicry-mediated autoimmune disorders [21], like systemic lupus erythematosus [22,23], hepatitis [24], and multiple sclerosis [25]. These pathogens possess proteins that mimic self-antigens, leading to cross-reactivity with host tissues. Additionally, molecular mimicry has been observed in viral infections, such as the hepatitis C virus, in which viral proteins share sequence homology with host proteins involved in autoimmune liver diseases [26].

Techniques that can be employed to study molecular-mimicking peptides include phage display [27], and bioinformatics approaches include sequence alignment and molecular modeling [28], etc. Sequence alignment algorithms can identify regions of similarity or shared motifs between the two protein sequences [29]. Homology modeling and comparative protein structure prediction can be used to analyze the three-dimensional structures [30] of pathogen and host proteins. By comparing the structural features and folding patterns, potential mimicking regions can be identified. Predictive algorithms, such as NetMHC [31] and the Immune Epitope Database (IEDB) [32], can be utilized to identify potential epitopes within pathogen proteins that resemble host epitopes associated with autoimmune disorders [33]. To validate the cross-reactivity of pathogen and host proteins, enzyme-linked immunosorbent assay (ELISA) assays [34], Western blotting [35], and flow cytometry [36], etc., can be employed. These methods measure the binding of antibodies or T cells to specific antigens and can confirm the presence of molecular mimicry. Apart from these, animal models, such as transgenic mice expressing human proteins associated with autoimmune disorders, can be used to study the effects of pathogen infections and evaluate the development of autoimmune responses [37,38]. Disease models, such as in vitro models of tissue inflammation can also be employed to investigate the consequences of molecular mimicry [8,39]. By utilizing a combination of these techniques, researchers can gain insights into the occurrence and mechanisms of molecular mimicry, contributing to a better understanding of its role in autoimmune disorders and potentially guiding the development of therapeutic interventions.

Bioinformatics is a swift approach to identifying and characterizing the molecular-mimicry interactions between human proteins and pathogens [40,41,42]. Various bioinformatics tools and databases are utilized to analyze protein sequences, identify shared motifs or structural similarities, and predict antigenicity and immunogenicity [43]. Comparative genomics and proteomics approaches are employed to identify pathogen proteins that mimic host antigens associated with autoimmune disorders [44,45,46]. Previous studies have utilized bioinformatics approaches to uncover potential molecular-mimicry mechanisms between pathogens and host proteins by employing sequence alignment algorithms, structural modeling, and epitope prediction tools to assess the extent of mimicry and the potential immunological consequences [15,41,47]. Additionally, database-mining techniques have been used to establish links between identified mimicry interactions and autoimmune disorders [9,48,49,50]. Herein, we analyzed mimicry prediction and association with autoimmune disorders in *Clostridioides difficile* using in silico methods. By integrating bioinformatics analyses with experimental validation, this information contributes to our understanding of the complex interactions between pathogens and the human immune system, shedding light on the role of molecular mimicry in the development and progression of autoimmune disorders.

## 2. Material and Methods

### 2.1. Homology Analysis

The entire set of proteins from the human and *C. difficile* S-0253 (reference strain ASM1888508v1) samples was obtained from Uniprot (https://www.uniprot.org/proteomes/UP000005640 (accessed on 31 May 2023)) and the NCBI database (GenBank accession: CP076401.1; accessed 31 May 2023), respectively. To identify potential homologous proteins, a local installation of BLAST was utilized, applying a threshold of ≥50% identity and ≥100-bit score to retain proteins for further analysis [16].

### 2.2. Mimic Region Identification

To identify potential regions involved in mimicry, the proteins were aligned to uncover regions of similarity with a minimum length of 10 amino acids [16]. RMSD was employed as a scoring metric to assess the structural similarity of the peptides. To obtain the 3D structures of these proteins, the state-of-the-art-predicted protein structures from the AlphaFold database were utilized [51,52]. To compare and align the obtained structures, both iPBA [53] and TM-align [54] algorithms were employed. These tools are widely recognized tools in the field of structural biology, known for their accuracy in comparing protein structures and determining alignment based on various structural features. iPBA is a sequence-independent method that uses a fragment-based approach (for capturing large protein fold changes) [53], while TM-align superimposes 3D coordinates and aligns protein structures by dynamic programming method (for capturing small fold changes) [54]. The superposed structures diagram was generated through the TM-align module of the RCSB PDB structural alignment tool (https://www.rcsb.org/alignment (accessed on 21 June 2023)).

### 2.3. Autoimmunity Elucidation

In order to identify homologous protein pathways associated with autoimmune disorders or infection, relevant databases, such as pathDIP [55] and PHAROS (https://pharos.nih.gov/targets/ (accessed on 15 June 2023)) [56], were surveyed, along with a thorough review of the literature. PHAROS provides preprocessed data from the Target Central Resource Database (TCRD) on the input of the human gene name, Uniprot ID, etc. Linked disorders can be manually checked for autoimmunity. For pathDIP, all databases were selected with a minimum confidence level set to 0.99. The data type selected was extended pathway associations. The protein interaction set considered for analysis included both experimentally detected and computationally predicted protein–protein interactions (PPIs) using the full IID dataset. pathDIP serves as a comprehensive reference for signaling cascades across various species, consolidating key pathways sourced from major curated pathway databases [57]. The associations in pathDIP are based on a combination of computational predictions, experimentally confirmed interactions, orthology mapping, and inference of physical protein interactions. This database provides a valuable resource for exploring and understanding signaling pathways associated with autoimmune disorders and infection. Apart from this, a literature search was conducted to identify infection or autoimmune pathways linked with these homologs. A BLAST search (≥90% homology) of epitopes was also carried out against IEDB [32], and the relevant literature was identified for the listed infection or autoimmune disorder in the database.

### 2.4. Mimic-Based Vaccine Design

Identified mimics were subjected to antigenic analysis using VaxiJen server [58]. Apart from this, properties like allergenicity, toxicity, and other parameters useful in finalizing peptides for vaccine design were studied. ProtParam was used for physicochemical profiling [59], while AllerCatPro (https://allercatpro.bii.a-star.edu.sg/, accessed on 16 June 2023) and ToxinPred (https://webs.iiitd.edu.in/raghava/toxinpred/multi_submit.php, accessed on 16 June 2023) were used for allergenicity and toxicity analysis, respectively. IEDB was used for immunogenicity prediction [32]. Mimics were prioritized for vaccine design based on these parameters, the ability to induce immune response, and conservation across the strains. Conservations was determined by the ConSurf webserver (https://consurf.tau.ac.il/consurf_index.php; accessed on 17 June 2023).

The vaccine construct was designed according to the previously described methodology [16], using suitable linkers, adjuvants, and binders. They were subjected to another round of evaluation according to properties like antigenicity, toxicity, allergenicity, etc. The best antigenic and non-allergenic, non-toxic construct was tested for immune reaction incitation and cloned in a pET-28(a)+ vector (available at https://www.snapgene.com/plasmids/pet_and_duet_vectors_(novagen)/pET-28a(%2B); accessed on 18 June 2023) after reverse translation and codon optimization through the JCat tool [60]. C-ImmSimm [61] was used for the simulation of immune reaction. The parameters were as follows: Simulation_volume = 10; Num_steps = 1000; HLA = A0101, A0102, B0702, B0704, DRB1_0101, DRB1_0102; No_of_injections = 3; Time of injection (in days) = 1, 30); Adjuvant = 100. The first two injections on day 1 and 30 were of the vaccine. At day 240, the proteins phosphoribosylaminoimidazolecarboxamide formyltransferase and adenylosuccinate lyase were injected to test the reaction.

### 2.5. Immune Receptor Binding Study

To elicit protection, the vaccine protein should bind the immune receptors with good affinity [62,63]. To analyze this property, we constructed a 3D model of the vaccine construct with SWISS-MODEL [64], AlphaFold [51], and I-TASSER [65]. The best model was selected based on Ramachandran plot statistics from the assess module of SWISS-MODEL (https://swissmodel.expasy.org/assess (accessed on 20 June 2023)) and energy-minimized using Molecular Operating Environment (MOE) v2016 software. It was then docked with immune receptors of importance like TLR-4 receptor (PDB ID: 3FXI), HLA-A (PDB ID: 3OX8), and HLA-B (PDB ID: 4JQX), using the ClusPro server [66]. ClusPro focuses on predicting the overall shape and orientation of the protein–protein complex [67]. Prodigy [68] was used to predict the thermodynamic properties and binding affinities of the obtained docked complexes. An experimentally determined protein–protein interacting complex (PDB ID:4GIQ) was employed as a control to compare predicted values. This comparison allowed us to assess whether the binding scores were superior or inferior to the control, providing valuable insights into the efficacy and specificity of our vaccine design approach.

## 3. Results

### 3.1. Homologous Sequence Identification

**Figure 1:**
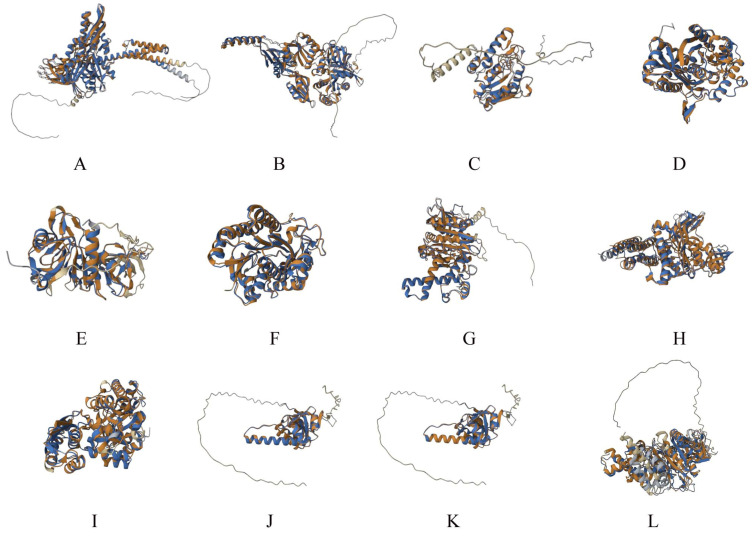
Superposed structures of the *C. difficile* and human homologous proteins. (**A**) Molecular chaperone DnaK; (**B**) Translation elongation factor 4; (**C**) Uracil-DNA glycosylase; (**D**) Acetyl-CoA C-acetyltransferase; (**E**) 3-oxoacid CoA-transferase subunit B; (**F**) UDP-glucose 4-epimerase GalE; (**G**) V-type proton ATPase subunit B; (**H**) V-type ATP synthase catalytic unit A; (**I**) Phosphopyruvate hydratase; (**J**) ATP-dependent Clp endopeptidase proteolytic subunit ClpP; (**K**) ATP-dependent Clp endopeptidase proteolytic subunit ClpP; (**L**) F0F1 ATP synthase subunit beta. Due to space constraints, the first 12 (Table 1) of the 23 proteins are shown here. Human homologs are shown in brown and bacterial proteins are shown in blue.

In total, 23 proteins were obtained with significant similarity between human and *C. difficile.* F0F1 ATP synthase subunit beta had the highest number of peptide mimics (*n* = 11), followed by F0F1 ATP synthase subunit alpha (*n* = 7) and heat-shock protein DnaK (*n* = 6) (Table 1). These were superposed (Figure 1), and RMSD after structural superposition varied between iPBA and TM-align prediction. A possible reason for this is the algorithm difference, in which iPBA is tailored for flexible proteins or regions within a protein. Thus, it gave overall lower RMSD values compared to TM-align. However, both servers gave the lowest RMSD of 0.51 for the ATP-dependent Clp endopeptidase proteolytic subunit ClpP. This suggests a high degree of structural similarity between the human and bacterial homologs of this protein.

### 3.2. Autoimmunity Prediction

PHAROS and PATHDIP database scan revealed several autoimmune diseases linked with the human homologous sequences of *C. difficile* (Table 2). ATP-dependent Clp protease proteolytic subunit and elongation factor Tu had two copies, so homologs were removed from database mining. For the rest of the homologs, the most commonly identified infection was tuberculosis (for DnaK, V-type ATP synthase, and ClpP) and the most commonly identified autoimmune disease was rheumatoid arthritis (for chaperones DnaK and GroEL, elongation factor tu, Translation elongation factor 4, 3-oxoacid CoA-transferase subunit B, V-type proton ATPase, phosphopyruvate hydratase, Phosphoribosylaminoimidazolecarboxamide formyltransferase, and NifU).

### 3.3. Sequence Mimics and Vaccine Design

Out of 68 mimics ≥ 10 amino acids in length, 31 were antigenic (Table 3), with four being allergenic. Among the 31 antigenic sequences, 14 mimics were identified as IL-4 inducers (RTTPSVVAFT, DHGKSTLADRL, GGAGYIGSHT, DGTGVRDYIHV, LGIYPAVDPL, IKEGDIVKRTG, CIYVAIGQKRST, IETQAGDVSAYIPTNVISITDGQI, EGHPDKICDQISD, TKVDRSAAYAAR, GAGQQSRIHCTRLAG, GCGSAIASSS, RGVKGTTGTQASFL, YKRNPMRSER). Only three peptides (PQIEVTFDIDANGIV, CIYVAIGQKRST, GAGQQSRIHCTRLAG), belonging to DnaK, F0F1 ATP synthase subunit alpha, and Adenylosuccinate lyase, were predicted as non-inducers of IL-6, while all the rest were inducers. Mimics LLLDVTPLSLGIET, DGTGVRDYIHV, VGERTREGNDLY, EGHPDKICDQISD, and RGVKGTTGTQASFL, belonging to DnaK, GalE, F0F1 ATP synthase subunit beta, methionine adenosyltransferase, and adenylosuccinate lyase, respectively, were predicted as inducers of IL-10.

**Figure 2:**
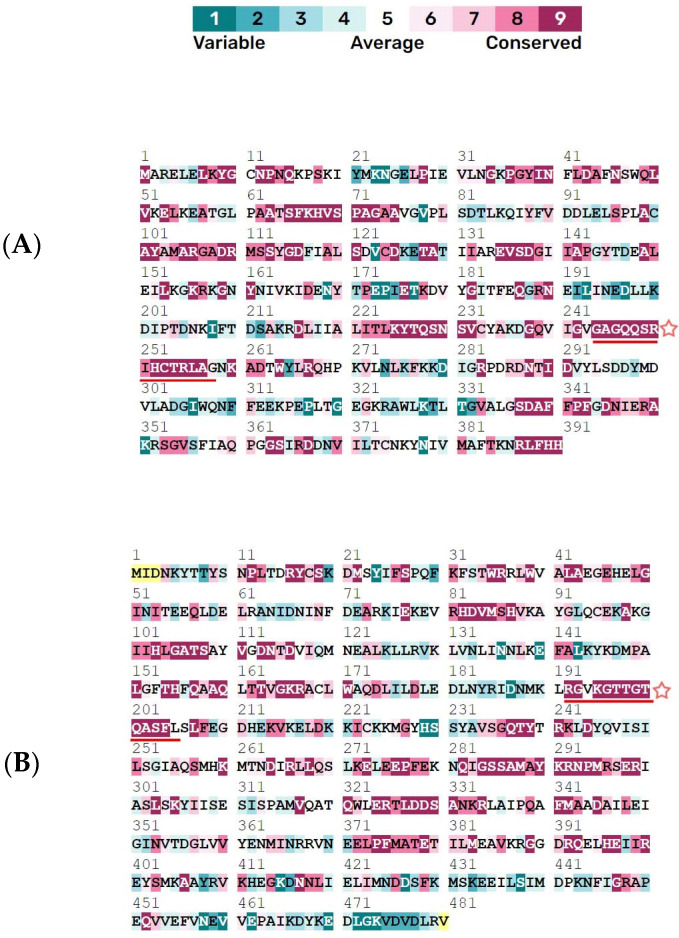
Conservation of sequence mimics from (**A**) phosphoribosylaminoimidazolecarboxamide formyltransferase and (**B**) adenylosuccinate lyase used for vaccine design underlined by red (and star symbol). Yellow color indicates insufficient data for conservation inference.

Two sequence mimics (GAGQQSRIHCTRLAG and RGVKGTTGTQASFL) belonging to the phosphoribosylaminoimidazolecarboxamide formyltransferase and adenylosuccinate lyase proteins, respectively, were selected for vaccine design based on antigenicity and other values. RGVKGTTGTQASFL was predicted as an inducer of IL-4, IL-6, and IL-10, while GAGQQSRIHCTRLAG was predicted as only an IL-4 inducer. Evolutionary analysis revealed majority of residues of both sequence mimics are highly conserved (Figure 2).

**Figure 3:**
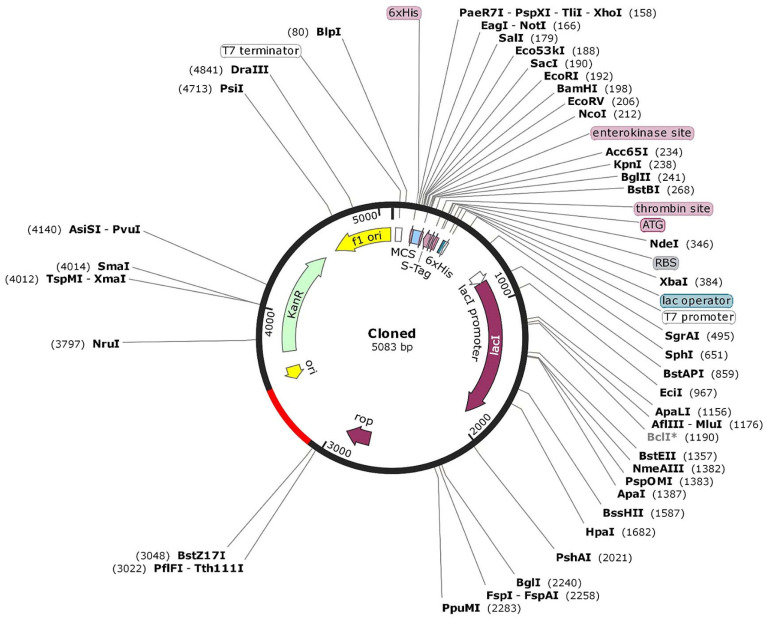
The 5083 bp cloned vector of the vaccine construct.

Mimic-based vaccine design is an innovative approach that utilizes synthetic peptides or proteins to mimic specific antigens of pathogens [16]. By presenting these mimics to the immune system, it can generate targeted immune responses against the actual pathogen. Here, in total, nine constructs were made, and the non-allergenic ones were retained for analysis (Supplementary Table S1). Among these, a stable, highly antigenic one, i.e., construct C2 was chosen for further downstream processing. It was reverse-translated and cloned in a pET-28(a)+ vector (Figure 3).

### 3.4. Immune Response Simulation

Immune response simulation analysis utilized Parker’s propensity scale to predict potential epitopes within the vaccine sequence [125], which may be recognized by the immune system, particularly by T cells. Six B-cell epitopes (EQIG, STRGRKCCRRKKEA, AGGGSRGVKGTTGT, AGGGSGAGQ, GGSHEY, AGGGS) were identified using the Parker propensity scale. For MHC-I, no binding epitope was present for the A0101 and B0702 allele, while two binding epitopes (IINTLQKYY and AGGGGSHEY) were identified for the A0102 allele and one (RVRGGRCAV) was identified for B0704. For MHC-II binding, seven epitopes were predicted for DRB1_0101 (YCRVRGGRC, FVAAWTLKA, WTLKAAAGG, LKAAAGGGS, VKGTTGTQA, FLGGGSAKF, LERAGAKFV) and two were predicted for DRB1_0102 (LKAAAGGGS and LERAGAKFV).

**Figure 4:**
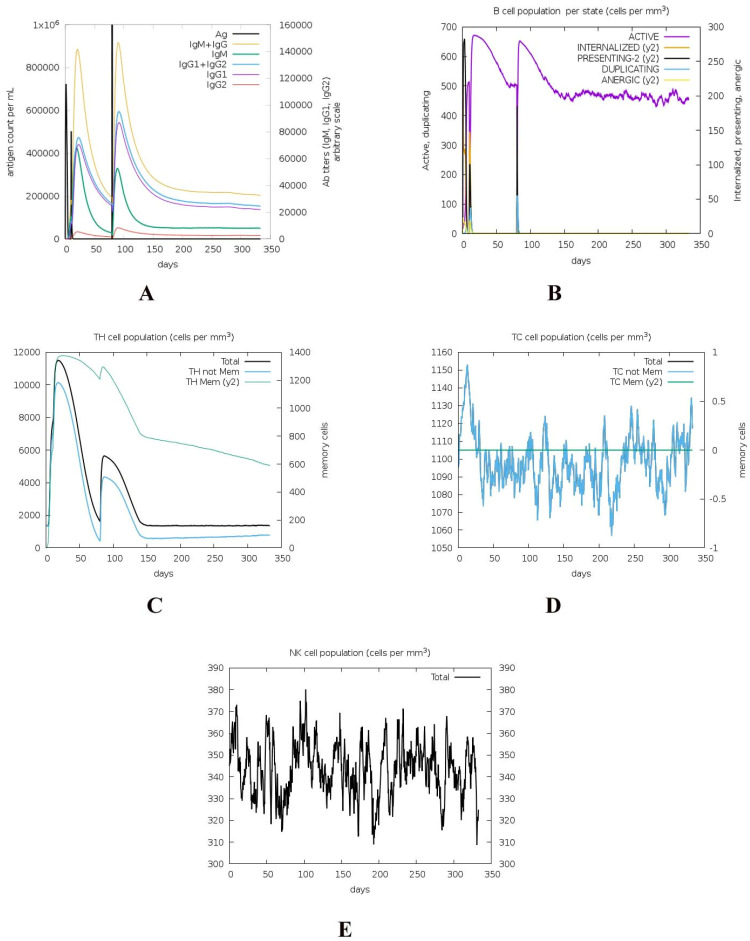
Immune system cells released after the C2 vaccine and *C. difficile* protein (phosphoribosylaminoimidazolecarboxamide formyltransferase and adenylosuccinate lyase) stress, including (**A**) immunoglobulins, (**B**) B cells, (**C**) TH cells, (**D**) TC cells, and (**E**) NK cells.

The IgM + IgG population was 140,000 cells/mm^3^, and only a slight difference was observed after the second injection (Figure 4A). This suggests that the primary immune response, characterized by the production of IgM antibodies followed by IgG antibodies [126,127], was already established after the first injection. The second injection did not result in a significant increase in the overall IgM + IgG population. IgG1 + IgG2 count was below 80,000 cells/mm^3^ but increased to more than 90,000 cells/mm^3^ after the second injection. This indicates that the secondary immune response, mediated by IgG antibodies [128], was robustly triggered by the second injection. The overall immune cell counts plateaued after around 200 days, indicating stabilization of the immune response. The B-cell population remained active after the vaccine injection, up to 100 cells/mm^3^ (Figure 4B). Their sustained presence suggests ongoing immune surveillance and the potential for long-term immune memory [129]. T-helper (TH) cells increased after each vaccine injection and remained active with a count of 4000 cells/mm^3^ even after 300 days (Figure 4C). TH cells play a crucial role in coordinating the immune response by facilitating communication between various immune cells, and their persistent presence indicates their continued involvement in supporting and regulating the immune response [130,131]. The count of cytotoxic T (TC) non-memory cells fluctuated, possibly indicating their active participation in eliminating target cells (Figure 4D). In contrast, TC memory cells remained consistently higher than 100 cells/mm^3^, suggesting the establishment of immunological memory. Memory cells enable a rapid and specific response upon re-exposure to the antigen, contributing to long-term immunity [132]. Natural killer (NK) cells have a role in innate immune defense [133], and their population remained at more than 300 cells/mm^3^ for the whole period (Figure 4E). No significant changes were observed in response to the stress of bacterial proteins. This suggests that the immune system reached a state of equilibrium and was no longer strongly influenced by the presence of bacterial proteins.

### 3.5. Vaccine Interaction

The 3D structure of the vaccine construct was modeled using three tools, in which SWISS-MODEL achieved the highest percentage (89.60%), indicating a larger portion of residues in favorable conformation compared to I-TASSER (44.32%) and AlphaFold (62.75%) (Supplementary Table S2). The QMEANDisCo Global score was additionally used to assess the global quality of the protein structure, with a lower score suggesting better overall quality. This metric is used to estimate the quality of a protein tertiary structure by taking distance constraints into account [134]. Again, SWISS-MODEL achieved a score of 0.64 ± 0.07, followed by AlphaFold (0.35 ± 0.07) and I-TASSER (0.32 ± 0.07).

**Figure 5:**
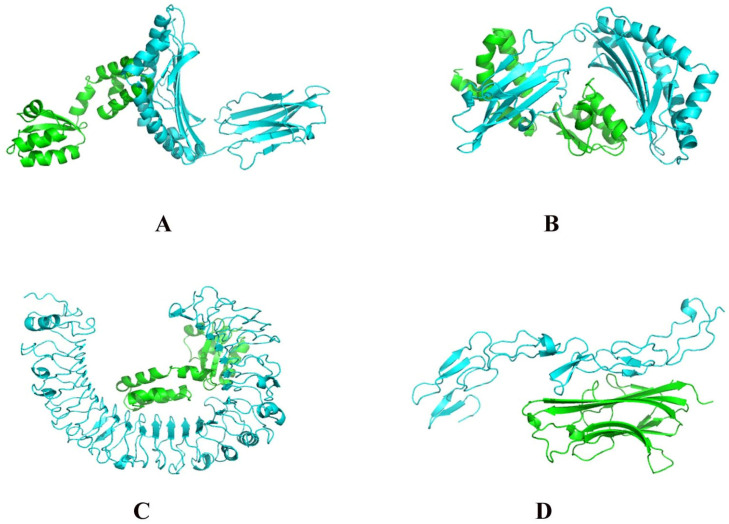
Vaccine construct (shown in green) interaction with (**A**) HLA-A, (**B**) HLA-B, and (**C**) TLR-4. Receptors are shown in cyan. (**D**) Control Tumor necrosis factor ligand superfamily member 11 (RANK-L) and 11A (RANK) from *Mus musculus*.

The best-modeled structure by SWISS-MODEL (Supplementary Figure S1A) was used to map interactions with HLA and TLR-4 receptor (Figure 5). Docking revealed that HLA-A and HLA-B complexes had relatively lower binding scores compared to the TLR-4 complex, implying stronger binding affinities between the vaccine construct and HLA receptors (Supplementary Table S3). The PRODIGY server [68] was used to map thermodynamic changes in these complexes, where ΔG (kcal mol^−1^) represents the change in free energy associated with the formation of the protein–protein complex, while K_d_ (M) provided the equilibrium dissociation constant at 25 °C. ΔG is studied to measure the stability of the complex, while K_d_ is studied to measure the binding affinity, with more negative values suggesting a stronger interaction [135,136]. ΔG and K_d_ are better predictors of binding than docking score [137] and were, therefore, employed for validation. HLA-B and TLR-4 had a highly negative ΔG value, indicating stable and stronger interaction compared to the control. TLR-4 indicated the lowest K_d_ value, suggesting a strong binding affinity and a favorable binding interaction in comparison with the control. This suggests that the interactions between the vaccine construct and TLR-4 receptor are likely to be more favorable and specific. This also shows that the ClusPro modeling method performed well in predicting the binding of the HLA and TLR-4 complex with vaccine construct and is a reliable approach to determine interactions.

## 4. Discussion

*C. difficile* can cause infections, primarily in the colon or large intestine [138,139]. Infection usually occurs in the immunocompromised [140] and in people who have received antibiotic therapy, when the natural balance of bacteria in the colon is disrupted [138]. This allows the bacterium to multiply and produce toxins that cause inflammation and damage to the intestinal lining. Molecular mimicry allows *C. difficile* toxin A to bind glycosphingolipids [141]. Mindur et al. have reported cross-reactive epitopes of myelin basic protein in the surface layer protein of a sub-species of *C. difficile* [142]. Peptide EQSLITVEGDKASM from the toxin B protein of the species has also been implicated in an autoimmune disease, namely primary biliary cirrhosis. Alam et al. have reported a collagen triple-helix repeat family protein in *C. difficile* as a mimic of the type II collagen protein of humans [143]. The protein is implicated in reactive arthritis, septic arthritis, and rheumatic symptoms. However, the sequence identity was less than 45%. This is why this protein was missed by our analysis, as we followed stringent criteria of identity value ≥50%.

The molecular mimics at the whole proteome scale for *C. difficile* and their involvement in autoimmune disorders have not yet been mapped. A bioinformatics-based approach is a useful method to exploit the publicly available data for this purpose. Mapping the molecular-mimicry mechanism employed by *C. difficile* can provide insights into the virulence and pathogenesis, as well as offer potential targets for the development of therapeutic interventions, such as vaccines or drugs that can disrupt the interaction between the bacterial mimics and the host receptors, thereby preventing or reducing the severity of *C. difficile* infections. For this purpose, we obtained the proteome of the reference strain of *C. difficile* S-0253 (*n* = 3732 proteins). Among these, only 23 proteins were homologous to humans, having ≥50% sequence identity. The structural superposition of these proteins revealed several regions with organizational and fold similarity (Table 1). The ATP-dependent Clp endopeptidase proteolytic subunit ClpP exhibited the lowest RMSD value, indicating a high degree of structural similarity between the human and bacterial homologs. It plays a crucial role in maintaining protein homeostasis in conjunction with chaperones by degrading misfolded or damaged proteins. The peptide sequence QIERDTERDRFLSAPEAV of *E. coli* ClpP has been previously implicated in autoimmune biliary liver cirrhosis [144]. The highest number of peptide mimics were observed in energy-generating F0F1 ATP synthase and heat-shock protein DnaK. Zhang et al. have reported increased activity of ATP synthase in the autoimmune neuromyelitis optica spectrum disorder [145]. DnaK has previously been implicated in molecular mimicry of other pathogenic bacteria like *Streptococcus pneumoniae* [16] and *Salmonella typhi* [15]. DnaK and other molecular chaperones like GroEL have been implicated in multiple autoimmune disorders [116,146,147,148,149,150,151]. DnaK has been associated with autoimmune atrophic gastritis caused by *H. pylori* [152], while Qeshmi et al. have reported its presence in multiple sclerosis as well [153]. Overall, rheumatoid arthritis, Alzheimer’s disease, psoriasis, Huntington’s disease, and Parkinson’s disease emerged as the primary autoimmune disease associated with multiple homologs.

Among infectious disease mapping, tuberculosis was the most common infection linked to the homologous proteins of *C. difficile,* suggesting a potential role of these proteins in the immune response against mycobacterial infections. A varying fraction of *C. difficile* infection in tuberculosis patients has been reported previously, ranging from ~3 cases per 1000 adults in Korea [154] to ~70 cases per 1000 individuals in South Africa [155]. Obuch-Woszczatyński reported *C. difficile*-mediated diarrhea in tuberculosis patients when rifampicin was used as part of their treatment regimen [156]. Rifampicin can contribute to the development of resistance against this antibiotic in *C. difficile*, which in turn poses a risk to the effectiveness of tuberculosis treatment. The rate of *C. difficile* infection in tuberculosis patients tends to be higher in aged people compared to younger adults [157]. This bacterium has also been identified as one of the predominant pathogens causing diarrheal illness in HIV-seropositive individuals, with two times higher prevalence compared to HIV-seronegative people [158].

Mimic-based vaccine design is an innovative approach that utilizes synthetic peptides or proteins to mimic specific antigens of pathogens [16]. By presenting these mimics to the immune system, targeted immune responses against the actual pathogen can be generated [159,160]. Hence, a stable and highly antigenic vaccine construct was designed using two peptide mimics identified in this study. It was cloned into a pET-28(a)+ vector and immune response was assessed using in silico simulations. The primary immune response, characterized by IgM production followed by IgG production, was established after the first vaccine injection. The secondary immune response, mediated by IgG antibodies, was robustly triggered by the second injection. The immune cell counts plateaued after approximately 200 days, indicating stabilization of the immune response. B cells remained active, suggesting ongoing immune surveillance and potential long-term immune memory. TH cells increased after each vaccine injection and remained active even after 300 days, indicating their continued involvement in supporting and regulating the immune response. TC cells showed fluctuating counts, possibly indicating their active participation in eliminating target cells, while TC memory cells remained consistently higher, indicating the establishment of immunological memory. NK cells, involved in innate immune defense, maintained a stable population throughout the study. Hence, a dynamic and robust immune response occurred following the vaccine injections. The presence of specific antibody populations sustained B-cell activity, and a stable count of T cells, NK cells, and EP cells indicated an effective immune response against the target antigen. The establishment of immunological memory and the plateauing of immune cell counts suggests a stable and functional immune system capable of long-term protection. No significant changes were observed in response to bacterial protein stress, suggesting that the immune system reached an equilibrium state and was no longer strongly influenced by the presence of bacterial proteins. However, computational predictions are not without limitations, and although mimic-based vaccine design and immune response simulation helps accelerate the vaccine-development process, providing insights into immune responses and generating hypotheses for further experimental investigations, it also has limitations. The foremost limitation is their accuracy and adverse response mapping due to inadequate input of variables and complexities of the immune system [16]. The local tissue microenvironment and factors such as blood flow, physical barriers, and cellular interactions can influence immune responses but may be overlooked or simplified in simulations. Additionally, the pathogens tend to mutate, and they may not be workable in the real-world scenario due to the altered genetics of the microbe. To overcome this, we have tried to focus on conserved epitopes of the antigenic proteins, but the immune evasion mechanism may be altered with time and the epitope may fail to generate an immune response. Thus, the in silico vaccine design is a valuable tool for narrowing down potential candidates and reducing research costs and timelines, but it is just the initial step in the vaccine-development process. Real laboratory-based testing is essential to validate and refine these designs, ensuring that the vaccine candidates are safe and effective in a real-world setting. Moreover, the in silico outcomes may not necessarily mirror those in a real laboratory as they typically do not consider the environmental factors the laboratory experiments take into account and can be overly optimistic or pessimistic predictions. Biological systems can also have unexpected interactions and feedback loops that are difficult to predict computationally. These interactions may only become apparent through real-world experimentation. The limitations of in silico modeling highlight the need for a comprehensive and rigorous approach to vaccine development that combines computational methods with empirical testing.

In summary, using bacterial peptides as structural templates for vaccine design is a valid approach, but the risk of triggering an autoimmune reaction prevails [43]. It is crucial to assess the risk associated with the potential induction of autoimmune response after administering the vaccine. It is also important to acknowledge that factors such as prior exposure to antigens and the presence of known autoantibodies, genetic predisposition of individuals, and other variables may contribute to the initiation of cross-reactive responses [161]. However, the immune system is normally fortified with multiple layers of protective mechanisms, which work in concordance to prevent the occurrence of autoimmunity [162] in response to vaccines. The mammalian immune system has also evolved an intricate repertoire of mechanisms to discern self- from non-self-antigens, primarily through the establishment of central immune tolerance [163,164], thus acting as a safeguard against autoimmunity. Moreover, molecular mimics, such as those utilized in vaccine design, tend to confer a reduced risk of provoking autoimmune reactions. This reduced risk can be attributed to the relatively lower immunological pressure imposed by these mimics when compared to actual pathogens, which inherently exert additional immune pressure due to the manifestation of the disease itself.

The regulatory T cells (Tregs) also play a pivotal role in modulating the immune response on encountering the antigen (from the vaccine), ensuring its proportionality and averting the development of autoimmunity [161]. These regulatory processes are further reinforced by natural checkpoints orchestrated by cytokines and other signaling molecules. These checkpoints serve as crucial regulators, fine-tuning the intensity and duration of the immune response [165], thus mitigating the risk of overly aggressive reactions that could harm the body tissues, instead of just the pathogen. Furthermore, cells can release immunosuppressive signals, including TGF-beta and IL-10, effectively dampening immune responses and preventing unwarranted reactivity against self-antigens [166,167]. Antigen-presenting cells (APCs) are another player in the immune system that present foreign antigens to immune cells, while self-antigens are less likely to incite a response [168]. As a result, the potential for cross-reactivity leading to autoimmune triggers is notably diminished in the context of mimic-based vaccine design, thus underscoring the safety and efficacy of this approach.

Additionally, the prudent approach of pre-vaccination testing for genetically predisposed individuals and the adoption of nanocarriers as alternatives to lipid adjuvants hold promise in mitigating the risk of cross-reactivity and triggering autoimmunity [163,169]. Furthermore, comparative evaluations of diverse vaccine formulations, concerning their capacity to induce or exacerbate pathology in relevant models, can yield valuable baseline data about the efficacy and safety of these vaccines. The inclusion of comprehensive immunological investigations, including autoimmune serology, within phases I to III of clinical trials is warranted to holistically assess vaccine responses. Hence, mimic-based innovative vaccine design, alongside the risk assessment and consideration of the inherent protective mechanisms of the immune system, offers a promising pathway toward vaccines that can effectively combat pathogens while sparing the human self-tissues from harm.

## 5. Conclusions

Investigation of autoimmune pathways associated with the identified human homologs of *C. difficile* revealed interesting connections to autoimmune diseases. The identified associations with autoimmune diseases, particularly rheumatoid arthritis, warrant further investigation into the underlying mechanisms of autoimmunity and the specific roles of these homologous proteins in disease pathogenesis. The structural similarity between human and *C. difficile* homologs suggests the possibility of using these bacterial proteins as structural templates for vaccine design and development. Understanding the conserved regions and functional motifs in these proteins may also aid in the design of therapeutics targeting *C. difficile* and related human diseases. We fabricated a vaccine construct using conserved, safe, and immunogenic mimics. It demonstrated good response in silico, but computational predictions have limitations, and we imply experimental research to complement or refute our findings.

## Figures and Tables

**Table 1:** RMSD of *C. difficile* homologs in humans. Molecular mimics with length ≥ 10 are also shown.

Serial no.	Name	UniProt ID of Human Homolog	NCBI Accession of Bacterial Homolog	Bacterial Protein Structure AlphaFold ID	No. of Similar Peptides (Length ≥ 10)	Molecular Mimic Region (Length ≥ 10)	Superposed Protein RMSD (iPBA)	TM-Align RMSD
1	Molecular chaperone DnaK	P38646	QWS53804.1	Q182E8	6	GIDLGTTNSCVAV RTTPSVVAFT INEPTAAALAYG LLLDVTPLSLGIET RNTTIPTKKSQ PQIEVTFDIDANGIV	1.13	2.0
2	Translation elongation factor 4	Q8N442	QWS53810.1	Q182F4	3	DHGKSTLADRL LNLIDTPGHVDF VLAKCYGGDI	1.25	1.55
3	Uracil-DNA glycosylase	P13051	QWS53824.1	Q182G9	0	-	1.01	1.13
4	Acetyl-CoA C-acetyltransferase	Q9BWD1	QWS54012.1	Q18AR0	1	NASGINDGAA	0.70	0.75
5	3-oxoacid CoA-transferase subunit B	E9PDW2	QWS54014.1	Q183B1	0	-	1.00	1.74
6	UDP-glucose 4-epimerase GalE	Q14376	QWS54050.1	Q183E8	3	GGAGYIGSHT VFSSSATVYG DGTGVRDYIHV	0.79	0.79
7	V-type proton ATPase subunit B	P21281	QWS54278.1	Q184E3	2	YAEALREVSAA THPIPDLTGYITEGQI	0.81	0.91
8	V-type ATP synthase catalytic unit A	P38606	QWS54279.1	Q184E7	2	MPVAAREASIYTGIT MADSTSRWAEALRE	0.98	1.03
9	Phosphopyruvate hydratase	P09104	QWS54488.1	Q181T5	3	LDSRGNPTVEV QEFMILPVGA VGDEGGFAPN	0.87	1.39
10	ATP-dependent Clp endopeptidase proteolytic subunit ClpP	Q16740	QWS54701.1	Q180F0	1	VVEQTGRGER	0.51	0.51
11	ATP-dependent Clp endopeptidase proteolytic subunit ClpP	Q16740	QWS54730.1	Q180J6	0	-	0.56	0.56
12	F0F1 ATP synthase subunit beta	P06576	QWS54823.1	Q184E3	11	TGIKVVDLLAPY KGGKIGLFGGAGVGKTVLI G VGERTREGNDLY GQMNEPPGAR DNIFRFTQAGSEVSALLGR PSAVGYQPTLAT TKKGSITSVQA YVPADDLTDPAPATTF LGIYPAVDPL LQDIIAILGMDELS RARKIQRFLSQ	2.11	2.73
13	F0F1 ATP synthase subunit alpha	P25705	QWS54825.1	Q184E7	7	IKEGDIVKRTG PIGRGQRELIIGDRQTGKTSI CIYVAIGQKRST YTIVVSATAS YDDLSKQAVAYR MSLLLRRPPGREAYPGDVFYLHSRLLERAAK IETQAGDVSAYIPTNVISITDGQI	1.52	2.69
14	Elongation factor Tu	P49411	QWS55098.1	Q18CE4	3	GTIGHVDHGKTTLTAAITK DCPGHADYVKNMITG DGPMPQTREH	0.98	1.12
15	Elongation factor Tu	P49411	QWS55112.1	Q18CE4	3	GTIGHVDHGKTTLTAAITK DCPGHADYVKNMITG DGPMPQTREH	0.98	1.12
16	Methionine adenosyltransferase	P31153	QWS55174.1	Q18CL7	4	EGHPDKICDQISD RFVIGGPQGD HGGGAFSGKD TKVDRSAAYAAR	0.72	0.93
17	Chaperonin GroEL	P10809	QWS55239.1	Q18CT5	3	AGDGTTTATVLA VVAVKAPGFGD DALNATRAAVEEGIV	1.22	3.61
18	Isocitrate/isopropylmalate dehydrogenase family protein	P50213	QWS55807.1	Q18A33	5	VTLIPGDGIGPE VMPNLYGDILSDL AGDGTTTATVLA VVAVKAPGFGD DALNATRAAVEEGIV	0.89	1.48
19	Phosphoribosylaminoimidazolecarboxamide formyltransferase	P31939	QWS55813.1	Q18A34	5	WQLVKELKEA SFKHVSPAGAAVG REVSDGIIAPGY KYTQSNSVCYAK GAGQQSRIHCTRLAG	0.73	1.23
20	Acyl-CoA dehydrogenase	P16219	QWS55947.1	Q18AQ1	2	LIFEDCRIPK ITEIYEGTSE	0.72	0.99
21	Acetyl-CoA C-acetyltransferase	Q9BWD1	QWS55952.1	Q18AR0	2	NASGINDGAA	0.70	0.75
22	Fe-S cluster assembly scaffold protein NifU	Q9H1K1	QWS56138.1	Q18BE3	2	GCGSAIASSS	1.01	1.39
23	Adenylosuccinate lyase	P30566	QWS56192.1	Q18BJ9	2	RGVKGTTGTQASFL YKRNPMRSER	0.81	0.94

**Table 2:** Autoimmune pathways of the selected homologs.

Serial no.		Protein Homolog	PHAROS		PATHDIP		Literature	
			Autoimmunity Pathway	Infection Pathway	Autoimmune Pathway	Infection Pathway	Autoimmune Pathway	Infection Pathway
1	P38646	Molecular chaperone DnaK	Autoimmune disease, Parkinson’s disease	Perinatal necrotizing enterocolitis, HIV, Tuberculosis	Parkinson’s disease, Huntington’s disease, Diabetes mellitus, Alzheimer’s	Tuberculosis, HIV, Papillomavirus, *E. coli*, Cytomegalovirus, *Staphylococcus* sp., Legionellosis, Chagas, Leishmaniasis, Measles	Guillain–Barré syndrome [69], Multiple sclerosis [70], Vitiligo [71], Systemic lupus erythematosus [72], Ankylosing spondylitis [73], Type I *Diabetes mellitus* [74], Rheumatoid arthritis [75]	Trypanosoma cruzi [76], *Mycobacterium leprae* [77]
2	Q8N442	Translation elongation factor 4	-	-		Tuberculosis, Rheumatoid arthritis	-	-
3	P13051	Uracil-DNA glycosylase	-	-	-	HIV, Viral carcinogenesis	-	-
4	Q9BWD1	Acetyl-CoA C-acetyltransferase	-	-	-	HBV, Viral carcinogenesis	Systemic lupus erythematosus [78]	HCV [79]
5	E9PDW2	3-oxoacid CoA-transferase subunit B	Crohn’s disease	-	-	-	Rheumatoid arthritis [80]	-
6	Q14376	UDP-glucose 4-epimerase GalE	Psoriasis, Interstitial cystitis	Tinea corporis, Tinea pedis	-	-	Type I Diabetes mellitus [81]	*Hemophilus influenzae* [82]
7	P21281	V-type proton ATPase subunit B	IgA glomerulonephritis	-	Huntington’s disease, Rheumatoid arthritis	*Helicobacter pylori* infection, HPV, Tuberculosis, Viral carcinogenesis, *Vibrio chloerae*, HIV	-	Tuberculosis [83], SARS-CoV-2 [84]
8	P38606	V-type ATP synthase catalytic unit A	Psoriasis, Myopathy	-	Alzheimer’s disease, Huntington’s disease, Parkinson’s disease, Rheumatoid arthritis,	HPV, *H. pylori*, Tuberculosis, *Vibrio cholerae*, Viral carcinogenesis, HIV	Thyroid eye disease [85]	Influenza H1N1 [86], Salmonellosis [87], Rabies virus [88], SARS-CoV-2 [89], Tuberculosis [90]
9	P09104	Phosphopyruvate hydratase	Psoriasis	-	-	-	Autoimmune encephalomyelitis [91], Rheumatoid arthritis [92]	Cytomegalovirus [93]
10	Q16740	ATP-dependent Clp endopeptidase proteolytic subunit ClpP	Psoriasis	-	Alzheimer’s disease, Huntington’s disease, Parkinson’s disease	-	-	Tuberculosis [94]
11	P06576	F0F1 ATP synthase subunit beta	-	-	Alzheimer’s, Huntington’s, Parkinson’s, Non-alcoholic fatty-acid liver diseases	Epstein–Barr virus infection, HBV, HCV, HPV, Measles, Legionellosis, *E. coli*	Autoimmune myocarditis [95]	MERS coronavirus [96], Echinococcus granulosus [97]
12	P25705	F0F1 ATP synthase subunit alpha	Alzheimer’s disease	-	-	-	Sjogren’s syndrome [98], Crohn’s disease [98], Ankolysing spondolytis [73]	-
13	P49411	Elongation factor Tu	-	-	Huntington’s, Parkinson’s	HCV, HBV, Legionellosis, *E. coli*, *V. cholerae*	Sjogren’s syndrome [99], Crohn’s disease [98], Ankolysing spondylitis [73]	*Streptococcus pneumoniae* [100], bacteria like *Bacillus anthracis*, *Francisella talurensis*, *Staphylococcus* sp., *E. coli*, *H. pylori*, etc. [101]
14	P31153	Methionine adenosyltransferase	Type 2 diabetes mellitus, demyelinating diseases, MODY, Psoriasis, fatty liver, or non-alcoholic steatohepatitis	-	-	-	Rheumatoid arthritis [102], Uveitis [103]	Herpes simplex type 1 [104], Poxvirus [105], West Nile virus [106]
15	P10809	Chaperonin GroEL	Allergic rhinitis	Tuberculosis, HIV	-	-	Type I Diabetes [103], Juvenile chronic arthritis [107], Atherosclerosis [108], Crohn’s disease [109], Rheumatoid arthritis [110], Systemic lupus erythematosus [111], Sjogren syndrome [112], Hashimoto thyroiditis [113,114], and myasthenia gravis [115], Autism [116]	*H. pylori* [117], *P. aeruginosa* and *S. aureus* [118]
16	P50213	Isocitrate dehydrogenase [NAD] subunit alpha	Psoriasis	-	Huntington’s disease, Parkinson’s disease	Epstein–Barr virus, HCV, Legionellosis	Atherosclerosis [119], Type I diabetes [120]	*H. pylori* [117]
17	P31939	Phosphoribosylaminoimidazolecarboxamide formyltransferase	Rheumatoid arthritis, Psoriatic arthritis, Erythrodermic psoriasis, Pustular psoriasis, Plaque psoriasis, Diabetes mellitus, Juvenile idiopathic arthritis	-	-	-	-	*C. neoformans* [121]
18	P16219	Acyl-CoA dehydrogenase	Allergic rhinitis, Ulcerative colitis, Crohn’s disease	-	-	-	-	-
19	Q9BWD1	Acetyl-CoA C-acetyltransferase	-	-	Parkinson’s disease	HBV, Viral carcinogenesis	-	-
20	Q9H1K1	Fe-S cluster assembly scaffold protein NifU	-	-	Parkinson’s disease	Influenza, HIV	-	Human respiratory syncytial virus [122], SARS-CoV-1 [96]
21	P30566	Adenylosuccinate lyase	Psoriasis	-	-	-	-	Schistosomiasis [123], *Chlamydia* sp. [124]

**Table 3:** Immunogenicity, allergenicity, and other properties of the antigenic mimics.

Serial no.	Antigenic Score	Sequence	Length	Immunogenicity Score	SVM Score for Toxicity	Hydrophobicity	Hydropathicity	Hydrophilicity	Charge	Mol Wt	Allergenicity
1.	1.09	GIDLGTTNSCVAV	15	0.65	0.07	0.85	−0.42	−1	1249.59	0.07	No
2.	0.58	RTTPSVVAFT	12	0.35	−0.07	0.4	−0.39	1	1078.36	−0.07	No
3.	0.89	LLLDVTPLSLGIET	12	0.31	0.15	1.18	−0.49	−2	1483.99	0.15	yes
4.	1.39	PQIEVTFDIDANGIV	11	0.29	0.04	0.42	−0.16	−3	1631.04	0.04	yes
5.	0.80	DHGKSTLADRL	12	0.26	−0.33	−1.01	0.66	0.5	1212.48	−0.33	No
6.	1.22	NASGINDGAA	12	0.26	−0.05	−0.22	0.04	−1	889.02	−0.05	No
7.	0.65	GGAGYIGSHT	24	0.21	0.08	−0.13	−0.52	0.5	919.11	0.08	No
8.	1.47	DGTGVRDYIHV	10	0.18	−0.15	−0.42	0.09	−0.5	1231.49	−0.15	No
9.	1.20	LDSRGNPTVEV	10	0.18	−0.23	−0.57	0.39	−1	1186.44	−0.23	yes
10.	1.48	VVEQTGRGER	11	0.14	−0.42	−1.26	0.88	0	1130.37	−0.42	No
11.	0.54	TGIKVVDLLAPY	10	0.12	0.1	0.91	−0.47	0	1288.73	0.1	No
12.	1.25	VGERTREGNDLY	11	0.12	−0.41	−1.48	0.77	−1	1408.66	−0.41	No
13.	1.03	PSAVGYQPTLAT	10	0.11	0.03	0.08	−0.58	0	1204.51	0.03	No
14.	1.04	TKKGSITSVQA	14	0.11	−0.19	−0.38	0.2	2	1119.44	−0.19	no
15.	0.54	LGIYPAVDPL	10	0.09	0.19	0.97	−0.67	−1	1057.4	0.19	yes
16.	0.65	IKEGDIVKRTG	10	0.06	−0.29	−0.69	0.86	1	1215.58	−0.29	No
17.	0.70	CIYVAIGQKRST	12	0.05	−0.13	0.2	−0.23	2	1338.76	−0.13	No
18.	0.53	YTIVVSATAS	10	0.02	0.14	1.22	−0.83	0	1011.27	0.14	No
19.	0.84	IETQAGDVSAYIPTNVISITDGQI	12	0.01	0.02	0.25	−0.26	−3	2506.13	0.02	No
20.	0.56	DGPMPQTREH	13	−0.05	−0.41	−2.06	0.7	−0.5	1167.4	−0.41	No
21.	1.41	EGHPDKICDQISD	10	−0.05	−0.28	−1.22	0.8	−2.5	1456.73	−0.28	No
22.	1.22	HGGGAFSGKD	12	−0.06	−0.1	−0.84	0.28	0.5	932.1	−0.1	No
23.	0.57	TKVDRSAAYAAR	15	−0.12	−0.35	−0.65	0.51	2	1308.6	−0.35	No
24.	1.75	AGDGTTTATVLA	12	−0.13	0.06	0.52	−0.28	−1	1077.32	0.06	No
25.	1.07	VTLIPGDGIGPE	10	−0.16	0.11	0.41	−0.11	−2	1167.51	0.11	No
26.	1.76	AGDGTTTATVLA	14	−0.17	0.06	0.52	−0.28	−1	1077.32	0.06	No
27.	1.62	GAGQQSRIHCTRLAG	13	−0.18	−0.23	−0.5	0.01	2.5	1554.97	−0.23	No
28.	1.22	NASGINDGAA	10	−0.19	−0.05	−0.22	0.04	−1	889.02	−0.05	No
29.	1.42	GCGSAIASSS	10	−0.20	0.06	0.66	−0.26	0	839.01	0.06	No
30.	1.65	RGVKGTTGTQASFL	11	−0.22	−0.14	−0.24	−0.07	2	1422.81	−0.14	No
31.	0.55	YKRNPMRSER	11	−0.25	−0.77	−2.62	1.19	3	1336.66	−0.77	No

## Data Availability

All the data used or generated in this study are provided as an accession number or relevant information as tables in the manuscript.

## Supplementary Materials

The following supporting information can be downloaded at: https://www.mdpi.com/article/10.3390/microorganisms11092300/s1, Figure S1: (A) 3D structure of vaccine construct using Swiss-Model (B) Ramachandran plot of the model; Table S1: Non-allergenic vaccine constructs against *C. difficile*; Table S2: 3D structure statistics of the vaccine construct, using various tools; Table S3: HLA and TLR receptor interaction statistics with the designed vaccine construct. Non-bonded contacts can involve attractive forces, such as van der Waals interactions and hydrophobic interactions, or repulsive forces, such as steric clashes. No disulphide bond was detected in any interaction.
