# Supplementary material for: Mining Autoimmune-Disorder-Linked Molecular-Mimicry Candidates in Clostridioides difficile and Prospects of Mimic-Based Vaccine Design: An In Silico Approach
Source: Microorganisms. 2023 Sep 12;11(9):2300. doi: 10.3390/microorganisms11092300 (PMC10536613; doi:10.3390/microorganisms11092300)
Supplement: Supplementary file 1 [file microorganisms-11-02300-s001.zip › microorganisms-2511041-supplementary.pdf]

Supplementary Table S1. Non-allergenic vaccine constructs against *C. difficile*.

**Serial no.**	**Construct**	**Length**	**antigenic**	**stability**	**GRAVY**	**Molecular weight**
1	>C1<br>EAAAKMAENSNIDDIKAPLLAALGAADLALATVNELITNLRERAEETRRSRVEESRARLTKLQEDLPEQLTELREKFTAEELRKAAEGYLEAATSELVERGEAALERLRSQQSFEEVSARAEGYVDQAVELTQEALGTVASQVEGRAAKLVGIELEAAAKAKFVAAWTLKAAAGGGS**GAGQQSRIHCTRLAG**GGGSAKFVAAWTLKAAAGGGSRGVKGTTGTQASFLGGGS**HEYGAEALERAG**AKFVAAWTLKAAAGGGS	260	1.02	unstable	-0.25	26957.14
2	>C2<br>**EAAAKGIINTLQKYYCRVRGGRCAVLSCLPKEEQIGKCSTRGRKCCRRKKEAAAKAKFVAAWTLKAAAGGGSRGVKGTTGTQASFLGGGSAKFVAAWTLKAAAGGGSGAGQQSRIHCTRLAGGGGSHEYGAEALERAGAKFVAAWTLKAAAGGGS**	155	1.36	stable	-0.20	15607.91
3	>C3<br>EAAAKMAENPNIDDLPAPLLAALGAADLALATVNDLIANLRERAEETRAETRTRVEERRARLTKFQEDLPEQFIELRDKFTTEELRKAAEGYLEAATNRYNELVERGEAALQRLRSQTAFEDASARAEGYVDQAVELTQEALGTVASQTRAVGERAAKLVGIELEAAAKAKFVAAWTLKAAAGGGSRGVKGTTGTQASFLGGGSAKFVAAWTLKAAAGGGSGAGQQSRIHCTR	269	1.00	stable	-0.29	28075.36

	LAGGGGS**HEYGAEALERAG** AKFVAAWTLKAAAGGGS					
4	>C4 EAAAKMAENSNIDDIKAPLL AALGAADLALATVNELITNL RERAEETRRSRVEESRARLTK LQEDLPEQLTELREKFTAEEL RKAAEGYLEAATSELVERGE AALERLRSQQSFEEVSARAE GYVDQAVELTQEALGTVAS QVEGRAAKLVGIELEAAAK AKFVAAWTLKAAAGGGSA KFVAAWTLKAAAGGGSRGV KGTTGTQASFLGGGS**HEYG AEALERAG**GAGQQSRIHCT RLAGGGGS**HEYGAEALERA G**AKFVAAWTLKAAAGGGS	272	0.99	unstabl e	-0.276	28241.4 9
5	>C5 EAAAKMAQVINTNSLSLLTQ NNLNKSQSSLSSAIERLSSGL RINSAKDDAAGQAIANRFTS NIKGLTQASRNANDGISIAQ TTEGALNEINNNLQRVRELS VQATNGTNSDSDLKSIQDEI QQRLEEIDRVSNQTQFNGVK VLSQDNQMKIQVGANDGET ITIDLQKIDVKSLGLDGFNVE AAAKAKFVAAWTLKAAAG GGSRGVKGTTGTQASFLGGG SAKFVAAWTLKAAAGGGSG AGQQSRIHCTRLAGGGGS**H EYGAEALERAG**AKFVAAWT LKAAAGGGS	285	1.10	stable	-0.32	29350.5 4
6	>C6 EAAAKMAENPNIDDLPAPL LAALGAADLALATVNDLIA	269	1.00	stable	-0.29	28075.3 6

	NLRERAEETRAETRTRVEERRARLTKFQEDLPEQFIELRDKFTTEELRKAAEGYLEAATNRYNELVERGEAALQRLRSQTAFEDASARAEGYVDQAVELTQEALGTVASQTRAVGERAAKLVGIELEAAAKAKFVAAWTLKAAAGGGSGAGQQSRIHCTRLAGGGGSAKFVAAWTLKAAAGGGSRGVKGTTGTQASFLGGGS**HEYGAEALERAG**AKFVAAWTLKAAAGGGS					
7	>C7 EAAAKMAENSNIDDIKAPLLAALGAADLALATVNELITNLRERAEETRRSRVEESRARLTKLQEDLPEQLTELREKFTAEELRKAAEGYLEAATSELVERGEAALERLRSQQSFEEVSARAEGYVDQAVELTQEALGTVASQVEGRAAKLVGIELEAAAKAKFVAAWTLKAAAGGGSRGVKGTTGTQASFLGGGS**HEYGAEALERAG**GAGQQSRIHCTRLAGGGGS**HEYGAEALERAG**AKFVAAWTLKAAAGGGS	255	0.95	unstable	-0.33	26653.65

Supplementary Table S2. 3D structure statistics of the vaccine construct, using various tools.

**Tool**	**MolProbity score**	**Ramachandran favoured residues (%)**	**Ramachandran outliers (%)**	**QMEANDisCo Global**
**I-Tasser**	**4.55**	**44.32**	**30.68**	**0.32± 0.07**
**Alpha Fold**	**3.58**	**62.75**	**23.53**	**0.35 ± 0.07**
**Swiss-Model**	**2.27**	**89.60**	**3.20**	**0.64 ± 0.07**

Supplementary Table S3. HLA and TLR receptor interaction statistics with the designed vaccine construct. Non-bonded contacts can involve attractive forces, such as van der Waals interactions and hydrophobic interactions, or repulsive forces, such as steric clashes. No disulphide bond was detected in any interaction.

**Serial no.**	**PDB ID**	**Complex**	**ClusPro lowest docking score with C2**	**ΔG (kcal mol^{-1})**	**K_d (M) at °C**	**Non-bonded interactions**	**No. of hydrogen bonds in main chains**	**Salt bridges**
1.	3OX8	HLA-A-vaccine	-814.8	-10.5	2.1e-08	158	10	3
2.	4JQX	HLA-B-vaccine	-878.6	-14.8	1.3e-11	149	12	3
3.	3FXI	TLR-4-vaccine	-770.4	-17.1	2.9e-13	226	19	6
4.	4GIQ (control)	Rank-RankL	-878.4	-11.6	2.9e-09	162	12	4

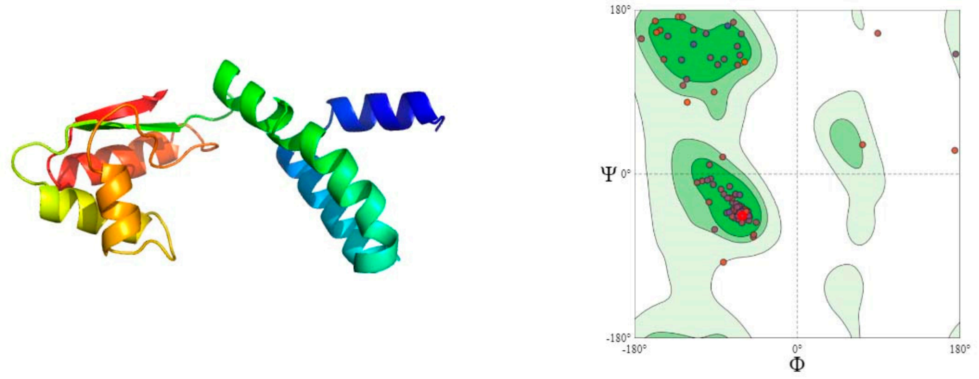

A B

Supplementary Figure S1(A) 3D structure of vaccine construct using Swiss-Model (B) Ramachandran plot of the model.
